# Myeloid-Derived Suppressor Cells in Tumors: From Mechanisms to Antigen Specificity and Microenvironmental Regulation

**DOI:** 10.3389/fimmu.2020.01371

**Published:** 2020-07-22

**Authors:** Yuhui Yang, Chunyan Li, Tao Liu, Xiaofang Dai, Alexandr V. Bazhin

**Affiliations:** ^1^Cancer Center, Union Hospital, Tongji Medical College, Huazhong University of Science and Technology, Wuhan, China; ^2^Department of Nuclear Medicine, Union Hospital, Tongji Medical College, Huazhong University of Science and Technology, Wuhan, China; ^3^Hubei Province Key Lab of Molecular Imaging, Union Hospital, Tongji Medical College, Huazhong University of Science and Technology, Wuhan, China; ^4^Department of General, Visceral, and Transplant Surgery, Ludwig-Maximilians-University Munich, Munich, Germany

**Keywords:** myeloid-derived suppressor cells, immune suppression, tumor microenvironment, immunotherapy, endoplasmic reticulum stress

## Abstract

Among the various immunological and non-immunological tumor-promoting activities of myeloid-derived suppressor cells (MDSCs), their immunosuppressive capacity remains a key hallmark. Effort in the past decade has provided us with a clearer view of the suppressive nature of MDSCs. More suppressive pathways have been identified, and their recognized targets have been expanded from T cells and natural killer (NK) cells to other immune cells. These novel mechanisms and targets afford MDSCs versatility in suppressing both innate and adaptive immunity. On the other hand, a better understanding of the regulation of their development and function has been unveiled. This intricate regulatory network, consisting of tumor cells, stromal cells, soluble mediators, and hostile physical conditions, reveals bi-directional crosstalk between MDSCs and the tumor microenvironment. In this article, we will review available information on how MDSCs exert their immunosuppressive function and how they are regulated in the tumor milieu. As MDSCs are a well-established obstacle to anti-tumor immunity, new insights in the potential synergistic combination of MDSC-targeted therapy and immunotherapy will be discussed.

## Introduction

Myeloid cells are a group of highly diverse cells that are essential for the normal functioning of innate and adaptive immunity. Mononuclear myeloid cells include monocytes, macrophages, and dendritic cells (DCs), and granulocytic myeloid cells include neutrophils, eosinophils, basophils, and mast cells. In steady state, myelopoiesis is under tight control and remains predominantly quiescent. A wide range of pathological stimuli, such as infectious microorganisms, tissue damage, and malignantly transformed cells, induce emergency myelopoiesis that largely leads to robust expansion of activated monocytes and neutrophils to eliminate potential threats. If these conditions terminate in time, the homeostasis of myeloid cells will be restored, leaving no negative consequence to the host; conversely, the persistent presence of low-strength stimuli leads to the accumulation of immature myeloid cells characterized by powerful immunosuppressive capacity, which may serve as a protective mechanism to prevent excessive tissue damage caused by unresolved immune response (1).

Studies since the 1970s have highlighted a group of systematically expanded and pathologically activated immature myeloid cells in tumor-bearing hosts. Based on their myeloid origin and immunosuppressive potency, these cells were termed myeloid-derived suppressor cells (MDSCs) in 2007 (2). In addition to cancer, MDSCs are implicated in other diseases, such as chronic inflammation or infection, autoimmune disorder, trauma, and graft-versus-host disease (2). MDSCs are a heterogeneous population consisting of myeloid progenitor cells and immature myeloid cells, characterized by the lack of surface markers associated with fully differentiated myeloid cells and by their morphological resemblance to granulocytic and monocytic cells (3).

MDSCs are generally divided into two main subsets: polymorphonuclear MDSCs (PMN-MDSCs, also known as granulocytic MDSCs) and monocytic MDSCs (M-MDSCs), which morphologically and phenotypically resemble neutrophils and monocytes, respectively. In tumor-bearing mice, MDSCs are generally defined as positive for myeloid lineage differentiation markers CD11b and Gr-1, with PMN-MDSCs being Ly6G^+^Ly6C^low^ and M-MDSCs being Ly6G^−^Ly6C^high^ (4). On the other hand, their counterparts in cancer patients are less definite, since studies on human MDSCs have been hampered by cellular diversity and a lack of unequivocal markers. Nonetheless, human PMN-MDSCs are now commonly defined as CD11b^+^CD14^−^CD15^+^ or CD11b^+^CD14^−^CD66b^+^ and M-MDSCs as CD11b^+^CD14^+^HLA-DR^−/low^CD15^−^ (4). Another population of immature MDSCs has recently been identified. These LIN^−^ (including CD3, CD14, CD15, CD19, and CD56) HLA-DR^−^CD33^+^ cells contain mixed groups of MDSCs comprising more immature progenitors and have been defined as “early-stage MDSCs (e-MDSCs)” (4). However, the murine equivalent of these e-MDSCs has not yet been defined.

**Figure 1:**
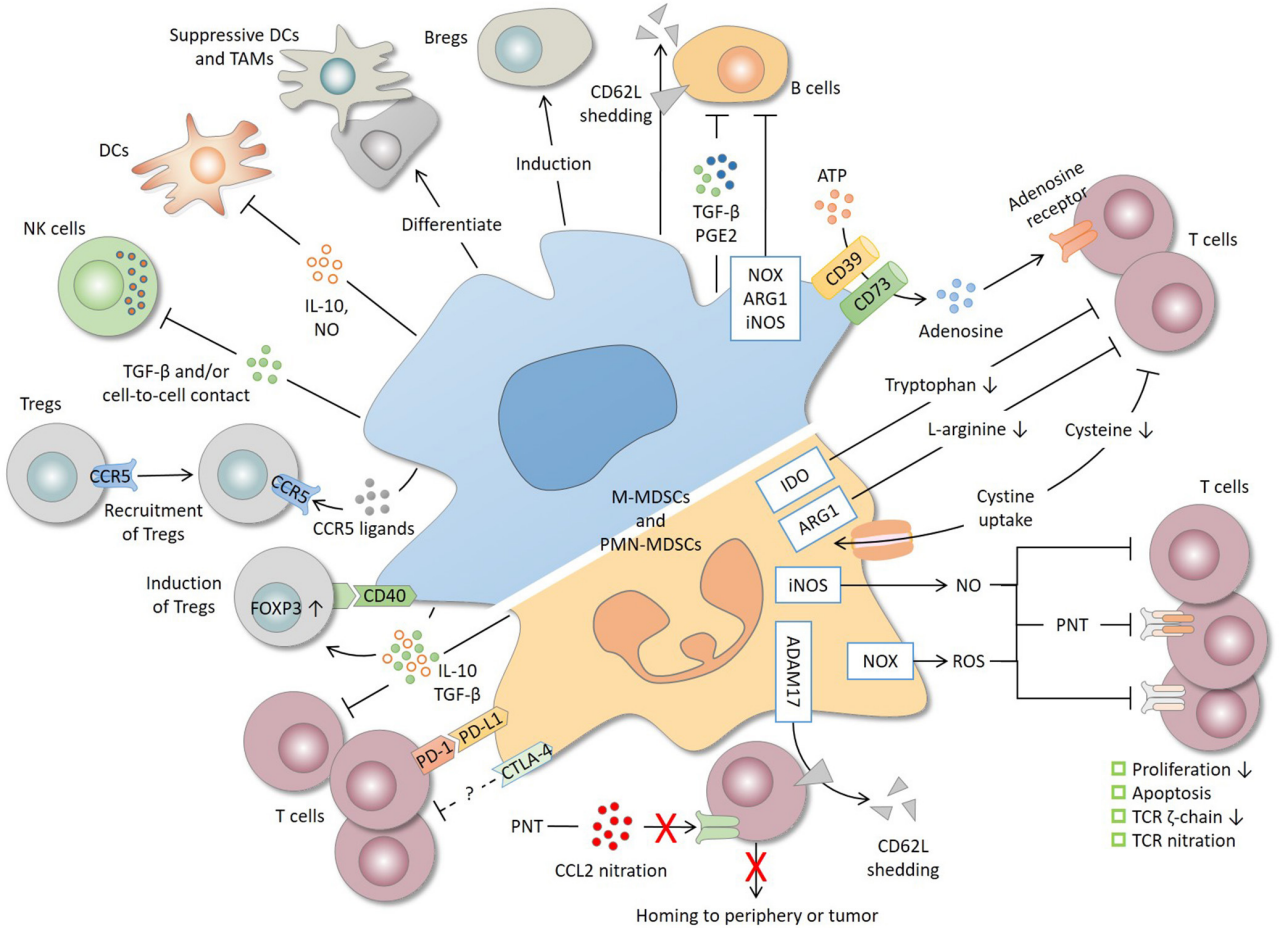
Immunosuppressive mechanisms and cell targets of MDSCs. T cells are the primary target of MDSCs. MDSCs produce a high level of nitric oxide (NO), reactive oxygen species (ROS), and peroxynitrite (PNT), which suppress T cells by inhibiting proliferation, inducing apoptosis, decreasing the TCR ζ-chain and nitrating the TCR complex. MDSCs deplete amino acids essential for T-cell response. For instance, MDSCs decrease L-arginine and tryptophan level through arginase 1 (ARG1) and indoleamine 2, 3-dioxygenase (IDO), respectively, and reduce the cysteine availability through cystine uptake. CD39/CD73 expression by MDSCs produces adenosine that inhibits T cells through adenosine receptors. By shedding CD62L (L-selectin) off the T-cell surface or by nitrating CCL2, MDSCs interrupt T-cell trafficking to the periphery or tumor site. MDSCs express both programmed cell death-ligand 1 (PD-L1), which inhibits T cells through interaction with programmed cell death protein 1 (PD-1), and cytotoxic T lymphocyte-associated antigen 4 (CTLA-4), whose precise role remains unclear. IL-10 and TGF-β, two major immunosuppressive cytokines produced by MDSCs, are implicated in T-cell suppression and regulatory T cell (Treg) induction. MDSCs also induce Tregs through CD40 in a contact-dependent manner and recruit Tregs through the production of various chemokines. In addition to T cells, MDSCs suppress natural killer (NK) cells and dendritic cells (DCs), inhibit B cells, and induce regulatory B cells (Bregs). Lastly, tumor-infiltrating MDSCs, mostly M-MDSCs, may differentiate into suppressive DCs and tumor-associated macrophages (TAMs). ADAM17, a disintegrin and metalloproteinase 17; iNOS, inducible nitric oxide synthase; NOX2, NADPH oxidase 2.

Activated MDSCs actively participate in multiple aspects of tumor progression, including immune evasion, angiogenesis, pre-metastatic niche formation, and epithelial-mesenchymal transition (EMT) (5–7). Among these tumor-promoting activities, suppression of immune cells is the defining feature of MDSCs. Since the aforementioned surface markers are not exclusive to MDSCs and some are shared by other myeloid cells, phenotyping together with suppressive function assessment has been proven to be the optimal strategy for identifying bona fide MDSCs (4). Studies in the past decade have provided us with a clearer view of the immunosuppressive nature of MDSCs. In this work, we intend to thoroughly review the ever-expanding list of suppressive machineries and cell targets of MDSCs (Figure 1). The nature of MDSC-mediated immune suppression will be discussed in detail, highlighting the antigen specificity of suppression and the regulatory role of the tumor microenvironment.

## Suppressive Mechanisms and Cell Targets of MDSCs

### Nitric Oxide, Reactive Oxygen Species, and Peroxynitrite

It is well-established that MDSCs are capable of inhibiting T-cell function. MDSCs express a high level of inducible nitric oxide synthase (iNOS), which produces nitric oxide (NO) (8–11). It is reported that NO suppresses T-cell proliferation, probably directly by inhibiting the Jak/STAT5 pathway or indirectly by inhibiting the antigen presentation from DCs (11, 12). Meanwhile, NO induces apoptosis of T cells (13). On the other hand, MDSCs produce a high amount of reactive oxygen species (ROS) via NADPH oxidase (NOX2) (8, 14). The inhibitory effect of ROS on T-cell function is well-described (15). For MDSCs, this suppression is caused by decreased expression of T-cell receptor (TCR) ζ-chain and is abrogated by inhibiting ROS production (14).

Studies have identified peroxynitrite (PNT), a potent oxidant produced by reaction between NO and superoxide anion (${\text{O}}_{2}^{\cdot -}$), as a crucial effector molecule of MDSCs. Local production of PNT in the tumor microenvironment is responsible for the non-responsiveness of tumor-infiltrating cytotoxic T lymphocytes (CTLs), and consistently, these CTLs are associated with a high level of nitrotyrosine, a marker of PNT activity (16). PNT suppresses T cells by nitrating the TCR complex, leading to loss of response to specific antigen presented by MDSCs (see below) (17). In addition to the TCR complex, it has recently been shown that MDSCs inhibit T-cell activation by nitrating Tyr394 of lymphocyte-specific protein tyrosine kinase (LCK), an initiating tyrosine kinase in the TCR-mediated signaling cascade (18).

### Interference With the Trafficking of T Cells

MDSCs impede the access of T cells to target sites by interfering with their trafficking (19). Expression of a disintegrin and metalloproteinase 17 (ADAM17), a major sheddase of L-selectin (CD62L), by MDSCs cleaves the ectodomain of L-selectin and consequently reduces L-selectin on the surface of naïve CD4^+^ and CD8^+^ T cells, therefore limiting their homing to peripheral lymph nodes and tumor sites (20). In another study, this MDSC-mediated decreased L-selectin level on T cells is regulated by high mobility group box protein 1 (HMGB1) in the tumor microenvironment (21).

Besides directly interfering with T-cell trafficking, MDSC-derived NO reduces E-selectin expression on endothelial cells, and PNT causes nitration and inactivation of CCL2 chemokine, both of which indirectly hamper the migration of T cells to the tumor site (22, 23).

### Depletion of Amino Acids Necessary for T-Cell Response

MDSCs are able to deplete amino acids required for T-cell activation and proliferation. A high level of arginase 1 (ARG1) expression by MDSCs depletes L-arginine in the tumor microenvironment, leading to downregulation of the CD3 ζ-chain of the TCR complex and proliferative arrest of T cells (24).

On the other hand, MDSCs deprive T cells of cysteine, an essential amino acid for T-cell activation, by uptaking cystine and not exporting cysteine. Since T cells depend on exogenously generated cysteine, the decreased availability of cysteine in the tumor milieu results in impaired T-cell activation (25). Furthermore, it is also reported that indoleamine 2, 3-dioxygenase (IDO) expression is upregulated in MDSCs isolated from fresh breast cancer tissue and is responsible for MDSC-mediated inhibition on T-cell proliferation and Th1 polarization (26).

### Adenosine and Adenosine Receptors

Recent studies have identified adenosine, a purine nucleoside, as a novel effector molecule of MDSCs. Extracellular ATP or ADP is hydrolyzed by CD39 (nucleoside triphosphate diphosphohydrolase) into AMP, which is in turn cleaved by CD73 (ecto-5'-nucleotidase) into adenosine (27). Both CD39 and CD73 are expressed by MDSCs from tumor-bearing mice and cancer patients, suggesting that MDSCs are capable of producing adenosine (28–30). TGF-β promotes the differentiation of MDSCs into CD39^+^CD73^+^ terminally differentiated myeloid cells with high adenosine production in tumor-bearing mice (31). Consistently, another recent study has demonstrated that tumor-derived TGF-β induces CD39/CD73 expression on MDSCs from lung cancer patients through the mammalian target of rapamycin (mTOR)-hypoxia-inducible factor 1α (HIF-1α) pathway, and these CD39^+^CD73^+^ MDSCs represent a distinct subpopulation that expresses higher levels of HIF-1α, cyclooxygenase 2 (COX2), IL-10, tumor necrosis factor (TNF)-α, and TGF-β as compared to their counterparts (32).

It is well-studied that adenosine inhibits the activation and effector function of T cells, which signals primarily through A_2A_ and A_3_ adenosine receptors (33). In the presence of CD73 substrate 5′-AMP, the inhibition of PMN-MDSCs on anti-CD3/CD28-induced T-cell proliferation is potentiated (28). On the contrary, CD73^−/−^ MDSCs or MDSCs whose CD39 or CD73 enzymatic activity is inhibited show reduced capacity to suppress T cells and natural killer (NK) cells (30, 32, 34). Furthermore, it is reported that MDSCs promote chemoresistance through the activity of CD39 and CD73 (32). Metformin, a biguanide used for type 2 diabetes, reduces the expression and activity of CD39 and CD73 on MDSCs, which leads to reduced MDSC-mediated suppression of CD8^+^ T cells *in vitro* and *in vivo*, and may partially account for the survival benefit seen in diabetic ovarian cancer patients treated with metformin (30).

The adenosine receptors expressed on MDSCs contribute indirectly to the adenosine-induced immune suppression. Stimulation of A_2B_ receptors preferentially expands PMN-MDSCs (28). In mice with melanoma, blockade of A_2B_ receptors reduces IL-10, monocyte chemoattractant protein 1 (MCP-1), and MDSCs in the tumor site, which is associated with increased frequency of intratumoral CD8^+^ T cells, elevated levels of TNF-α and IFN-γ, and delayed tumor growth (35). In another murine melanoma model, selective deletion of A_2A_ receptors in myeloid cells leads to significantly reduced IL-10 production by MDSCs, an increase in activated CD8^+^ T cells and NK cells, and delayed primary tumor growth and metastasis (36).

CD39 and CD73 are also expressed on tumor cells, regulatory T cells (Tregs), effector T cells, Th17 cells, and other stromal cells (33). Ectonucleotidases are supposed to prevent excessive T cell-mediated immune response and to regulate the balance between pro-inflammatory ATP and immunosuppressive adenosine. However, tumor hijacks this network to facilitate immune evasion. In line with the abovementioned findings, Umansky et al. have proposed two modes of adenosine signaling. Firstly, MDSCs, Tregs, and tumor cells may produce extracellular adenosine to suppress T-cell function in a paracrine manner. Secondly, adenosine produced by ectonucleotidase on tumor-infiltrating lymphocytes suppresses their own function in an autocrine manner; the upregulated CD39 and CD73 expression by MDSCs and Tregs also enables autocrine adenosine signaling and potentiates their expansion and/or suppressive activity (33).

### MDSC-Derived IL-10

MDSCs are a major source of IL-10 in tumor-bearing host (37–40), and consistently, the frequency of MDSCs is correlated with the IL-10 level in peripheral blood of cancer patients (41). It is becoming clear that IL-10 serves as a non-redundant suppressive mechanism of MDSCs, and accordingly, blockade of IL-10 signaling or neutralization of IL-10 leads to alleviated T-cell suppression, delayed tumor progression, and improved therapeutic efficacy (37, 42). In addition to T-cell inhibition, MDSC-derived IL-10 is implicated in the induction of Tregs and the suppression of DCs (see below).

Recent studies are unraveling the regulation on IL-10 production by MDSCs, which involves cellular and non-cellular participants. For instance, hypoxia significantly upregulates IL-10 secreted by MDSCs (43). Exposure to lipopolysaccharide (LPS), a Toll-like receptor (TLR) ligand, increases IL-10 production by MDSCs, which may require the MyD88 signaling pathway (44). Transmembrane TNF-α (tmTNF-α), but not the secretory form, activates MDSCs to upregulate IL-10 and other immunosuppressive effector molecules through TNFR2 (45). The level of interferon regulatory factor 4 (IRF4), an essential transcription factor required for lymphoid and myeloid cell differentiation, reduces remarkably during the development of MDSCs and modulates the suppression of T cells through IL-10 and ROS production (46). Tumor cells, not surprisingly, participate in the MDSC-derived IL-10 regulation. For instance, knockdown of semaphorin 4D, a pro-angiogenic factor overexpressed in many malignancies, in tumor cells reduces the IL-10 production by MDSCs (47). Glioma stem cell-derived exosomes induce systemic T-cell suppression by polarizing CD14^+^ monocytes toward M-MDSC phenotype with heightened IL-10 level (48). In another study, the NKG2D ligand RAE-1ε expressed on tumor cells facilitated the expansion and activation of MDSCs that display pronounced ARG1 activity and IL-10 production (49).

Similarly, MDSCs developed in the settings of microbial infection are also capable of producing IL-10 (50–52). In patients with chronic hepatitis B, IL-10 induced by programmed cell death protein 1 (PD-1) signaling is responsible for T-cell suppression by MDSCs (50). In patients with chronic hepatitis C virus infection, M-MDSCs have higher levels of phosphorylated STAT3 and IL-10, while blocking STAT3 signaling reduces hepatitis C virus (HCV)-mediated M-MDSC expansion and IL-10 expression (51).

### TGF-β

TGF-β is another well-documented immunosuppressive cytokine secreted by MDSCs in tumor-bearing host (22, 43, 53). MDSCs developed in non-cancer settings are also capable of producing TGF-β (52, 54). Evidence for the regulation of MDSC-derived TGF-β remains elusive. It was shown previously that TGF-β produced by MDSCs is induced *in vivo* by IL-13 and CD1d-restricted T cells that are most likely natural killer T (NKT) cells (55). Recent studies have shown that TGF-β production by MDSCs is regulated by tmTNF-α, ribosomal protein S19, and semaphorin 4D (45, 47, 56). On the contrary, CD14^+^HLA-DR^−/low^ MDSCs from patients with liver cancer show no TGF-β secretion (57). These findings suggest that TGF-β production by MDSCs may be context-dependent.

MDSC-derived TGF-β contributes to T-cell suppression, although it is probably not the principal mechanism (53). CD14^+^HLA-DR^−/low^ MDSCs isolated from melanoma patients inhibit T cells via TGF-β with no involvement of ARG1 and iNOS (58). Song et al. have shown that transfer of tumor-derived MDSCs to asthmatic mice leads to reduced pulmonary recruitment of inflammatory cells, suppressed Th2 response, and decreased IgE production in a TGF-β1-dependent manner (59). Furthermore, TGF-β is essential in Treg induction by MDSCs (see below).

Other immune cells are also inhibited by MDSC-derived TGF-β. For instance, in a murine model of AIDS, M-MDSCs suppressed B-cell response by superoxide, nitric oxide, PNT, and TGF-β (54). CD14^+^HLA-DR^−/low^ MDSCs from melanoma patients inhibit NK cells primarily through TGF-β that is stimulated by tumor-derived PGE2 (60). In addition to soluble TGF-β, MDSCs expanded in tumor-bearing mice express and utilize membrane-bound TGF-β to suppress NK cells and NKT cells in a contact-dependent manner (61, 62).

In addition to immune suppression, TGF-β has been implicated in the regulation of tumor metastasis facilitated by MDSCs. A portion of tumor cells undergoes EMT to disseminate, invade surrounding tissue, and metastasize. In a spontaneous murine model of melanoma, Toh and colleagues have shown for the first time that MDSCs use TGF-β, epidermal growth factor, and hepatocyte growth factor to induce EMT and that depletion of MDSCs results in reduced EMT and fewer metastases (63). In another study, anti-TGF-β treatment in a murine model of mammary tumor inhibited tumor growth and lung metastasis, and depletion of MDSCs diminished this beneficial effect of TGF-β neutralization (64). Another study from the same group later demonstrated that specific deletion of gene encoding TGF-β receptor II in myeloid cells significantly reduces metastasis, which is mediated by decreased TGF-β1 and type 2 cytokine production and by reduced ARG1 and iNOS expression. This effect was largely ascribed to the CD11b^+^Ly6G^+^ myeloid subset (65).

### PD-L1 and CTLA-4 Expression by MDSCs

Immune checkpoint pathways act as negative regulators and prevent excessive immune response. MDSCs assist tumor to hijack this mechanism in order to promote T-cell anergy, which signals mostly through the PD-1/programmed cell death-ligand 1 (PD-L1) pathway (66). MDSCs express PD-L1 in various tumor models (43, 67–73). Meanwhile, numerous studies have found PD-L1 expression in MDSCs from cancer patients (29, 42, 53, 72, 74–76). In liver cancer patients, the percentage of PD-L1^+^ MDSCs in peripheral blood correlates with disease stage and correlates inversely with clinical outcome (76). On the other hand, MDSCs developed during microbial infection also express PD-L1 (77, 78).

PD-L1 is implicated in MDSC-mediated T-cell suppression. PD-L1 blockade reduces the suppressive capacity of MDSCs on T cells (29, 42, 53, 68, 73, 74, 77–79). In addition to conventional T cells, in a murine model of liver metastasis, PD-L1 expression by MDSCs impairs the proliferation of chimeric antigen receptor cells, while MDSC depletion or PD-L1 blockade improves their therapeutic efficacy (80). Blocking PD-L1 relieves inhibition on DCs by MDSCs as well (81).

Several studies have shown that tumor-infiltrating MDSCs express a higher level of PD-L1 than their peripheral counterparts, suggesting microenvironmental regulation of PD-L1 expression (43, 68, 72, 73, 75). For instance, tumor cells upregulate the PD-L1 expression in MDSCs by interfering with their arachidonic acid metabolism (82). Tumor-derived soluble mediators are also responsible for PD-L1 induction in intratumoral MDSCs (76, 80). Other microenvironmental signals that regulate PD-L1 expression by MDSCs, such as hypoxia, cytokines, and stromal cells, will be discussed in detail in the following sections.

On the other hand, it is reported that MDSCs express cytotoxic T lymphocyte-associated antigen 4 (CTLA-4) (43, 71). However, unlike PD-L1, the precise role and regulation of CTLA-4 is less well-studied in MDSCs. It is reported that blocking or silencing CTLA-4 reduces the frequency and ARG1 activity of MDSCs (83).

### Induction and Recruitment of Regulatory T Cells

MDSCs inhibit effector T cells not only by themselves but also by inducing and recruiting Tregs. The proliferation of Tregs is relatively insensitive to suppression by MDSCs as compared with effector T cells (84). Intratumoral accumulation of Tregs occurs later than that of MDSCs, while depletion of MDSCs reduces infiltrating Tregs, suggesting that MDSCs may facilitate the development of Tregs (85). In non-cancer settings, co-culturing CD4^+^ T cells with MDSCs from HIV^+^ individuals or chronic hepatitis C patients significantly increases the differentiation of Foxp3^+^ Tregs (51, 86).

The mechanism(s) for Treg induction by MDSCs is not fully understood. During tumor progression, a subset of DCs with an immature myeloid phenotype is licensed by tumor cells to promote proliferation of Tregs by producing TGF-β (87). Huang and colleagues have shown that MDSCs induce Tregs both *in vitro* and *in vivo*, which requires activation of T cells and is dependent on IFN-γ and IL-10. The authors speculated that, in response to IFN-γ produced by activated T cells, MDSCs secret TGF-β and IL-10, both of which participate in the development of Tregs (88). Another study from this same group later demonstrated that CD40 expression on MDSCs is required for Treg induction, since adoptive transfer of CD40-deficient MDSCs or administration of anti-CD40 antibodies fails to induce Tregs (89). Treg induction by MDSCs is attenuated in the Transwell system that separates the two cell types, suggesting the requirement of direct cell-to-cell contact (90). In a murine model of B-cell lymphoma, MDSCs promoted the expansion of Tregs from pre-existing natural Tregs but not conversion from naïve T cells. In that study, MDSCs induced tumor-specific Tregs via antigen uptake, processing, and presentation, which requires ARG1 but not TGF-β (91).

In addition, MDSCs may promote the recruitment of Tregs to the tumor milieu. Tumor-infiltrating M-MDSCs produce CCR5 ligands CCL3, CCL4, and CCL5, and meanwhile, Tregs exhibit high surface expression of CCR5 and are recruited to tumor tissue by CCL4 and CCL5. Accordingly, Tregs from CCR5 knockout mice almost completely lost their ability to migrate toward M-MDSCs *in vitro* (92). In a murine model of glioblastoma multiforme, both M-MDSCs and Tregs were recruited by CCL2 produced by tumor-associated macrophages (TAMs) and microglia (93). A recent study revealed a closed loop between mast cells, MDSCs, and Tregs in the tumor microenvironment. Mast cells induce infiltration of MDSCs to tumor and induce their IL-17 secretion; MDSC-derived IL-17 attracts Tregs indirectly and potentiates their suppressive activity and IL-9 production; IL-9 in turn promotes the survival and tumor-promoting function of mast cells. In that study, IL-17 promoted Treg recruitment by increasing the level of CCL17 and CCL22 in the tumor microenvironment (94).

Studies on the relation between MDSCs and Tregs in cancer patients are relatively limited. A positive correlation between MDSCs and Tregs in peripheral blood and tumor site has been detected in cancer patients (40, 95). Hoechst and colleagues have shown that CD14^+^HLA-DR^−/low^ M-MDSCs from hepatocellular carcinoma patients induce suppressive CD4^+^CD25^+^Foxp3^+^ Tregs in a contact-dependent manner when co-cultured with autologous CD3/CD28-stimulated CD4^+^ T cells (57). In addition, to induce Tregs from CD4^+^ T cells, a study from the same group has shown that CD14^+^HLA-DR^−/low^ M-MDSCs are able to convert Th17 cells to Foxp3^+^ Tregs, which is dependent on MDSC-derived TGF-β and retinoic acid (96). Jitschin et al. have shown that M-MDSCs from chronic lymphocytic leukemia (CLL) patients suppress T-cell activation and promote Treg induction, which is partly dependent on IDO activity (95). Furthermore, the authors have also demonstrated that after co-culture with CLL cells, monocytes from healthy donors resemble the phenotypic, suppressive, and Treg-inducing characteristics of M-MDSCs from CLL patients (95). In patients with lung cancer, a novel tumor-infiltrating B7-H3^+^CD14^+^HLA-DR^−/low^ subset of MDSCs is reported to induce Tregs *in vitro*, which is partly dependent upon IL-10 (40).

Interestingly, there are also reports revealing no clear association between MDSCs and Tregs. In mice bearing T-cell lymphoma, the percentage of intratumoral Tregs is invariably high throughout tumor growth and does not relate to the accumulation kinetics of MDSCs (9). In another study, the T-cell non-responsiveness induced by adoptive transfer of MDSCs was not caused by Treg induction (97). Furthermore, in contrast to the abovementioned Treg-inducing action of M-MDSCs, it is reported that PMN-MDSCs impair TGF-β-mediated generation of inducible Tregs (iTregs) from naïve T cells and inhibit proliferation of naturally occurring Tregs (nTregs) without affecting Foxp3 expression (98). These discrepancies need to be clarified by further study.

### Suppression of Natural Killer Cells

NK cells are another major target of MDSCs. The reduced number and impaired function of NK cells in tumor-bearing mice are inversely correlated with the increased level of MDSCs and are restored by depletion of MDSCs (61, 99). A similar inverse correlation is also observed in patients with non-Hodgkin lymphoma (39). It is shown that the enhanced lactate production by tumor cells inhibits NK cells not only directly by inhibiting their cytotoxicity but also indirectly by increasing the number of MDSCs (100). Interestingly, a recent study has demonstrated that a portion of immature NK cells is converted into MDSCs in the presence of GM-CSF and that this conversion is abolished by IL-2 exposure (101). This novel developmental pathway of MDSCs may account, at least partially, for the reduced level of NK cells in tumor-bearing host.

In murine models, the cytotoxicity, NKG2D expression, and IFN-γ production of NK cells are inhibited by MDSCs both *in vitro* and *in vivo* (61, 102). This suppression is contact-dependent and requires membrane-bound TGF-β1 on MDSCs (61, 102). In a recent study, Elkabets et al. identified a novel subset of Gr-1^high^ PMN-MDSCs that is induced by IL-1β and lacks Ly6C expression (Ly6C^neg^). These Ly6C^neg^ MDSCs produce higher levels of iNOS and ROS than Ly6C^low^ MDSCs and, correspondingly, exhibit stronger suppression of T cells and NK cells (103). The MDSC-mediated NK cell suppression is associated with increased metastasis in mice during gestation (104). In tumor-bearing mice treated with medroxyprogesterone acetate, which is commonly used as hormone replacement therapy and as a contraceptive, MDSCs exhibit higher suppression of NK cells as compared with MDSCs from control mice, implying a potential mechanism for increased breast cancer incidence associated with prolonged medroxyprogesterone acetate administration (105).

In patients with liver cancer or advanced melanoma, CD14^+^HLA-DR^−/low^ MDSCs suppress autologous NK-cell cytotoxicity and IFN-γ production (60, 106). This suppression is independent of ARG1 and iNOS but requires cell-to-cell contact through NK-activating receptor NKp30 on NK cells, suggesting expression of NKp30 ligand(s) by MDSCs (106). In addition, TGF-β produced by MDSCs from melanoma patients, which is stimulated by PGE2, also serves as a major mechanism for NK-cell suppression (60). In addition, MDSCs from cancer patients inhibit Fc receptor-mediated signal transduction and downstream effector function of NK cells, including antibody-dependent cellular cytotoxicity and cytokine production, probably through NO production (107).

As an essential defensive mechanism of the innate immune system, it is not surprising that NK cells are suppressed by MDSCs generated in microbial infection. It is shown that polymorphonuclear neutrophils and PMN-MDSCs dampen the activation and cytotoxic activity of NK cells toward *Aspergillus fumigatus* (108). In another study with mice infected by vaccinia virus, PMN-MDSCs negatively regulated the proliferation, activation, and function of NK cells, which helped to contain excessive NK cell activity (109). In HCV infection, CD33^+^CD11b^low^HLA-DR^low^ MDSCs suppress the IFN-γ production of NK cells by depleting L-arginine via ARG1 (110). Interestingly, CD66b^+^CD33b^+^HLA-DR^low^ PMN-MDSCs increase strikingly in the cord blood of neonates when compared with peripheral blood of healthy children and adults. These cord blood PMN-MDSCs are able to inhibit the function of T cells and NK cells, which may be responsible for the impaired host defense of neonates (111).

Conversely, there are studies showing NK cell activation by MDSCs. For instance, Nausch et al. have found that MDSCs from tumor-bearing mice express NKG2D ligand RAE-1 and activate NK cells to produce IFN-γ, which is partially contact-dependent and requires signaling through NKG2D (112). In mice bearing NK-sensitive tumor, poly I:C treatment allows MDSCs to prime NK cells and consequently leads to delayed tumor growth. MDSC-derived IFN-α after poly I:C administration activates NK cells, which drives CD69 expression and IFN-γ production but does not induce cytotoxic activity of NK cells (113). A recent study has shown that M-MDSCs infiltrate in the tumor microenvironment prior to NK cells and are required for the tumoricidal activity of NK cells to eradicate galectin-1-deficient GL26 glioma (114). Taken together, the seemingly contradictory findings mentioned above suggest that the effect of MDSCs on NK cells, either inhibitory or stimulatory, is most likely context-dependent.

### Impaired Function of Dendritic Cells by MDSCs

Relatively less information is available on the direct impact of MDSCs on DCs. Accumulation of MDSCs in tumor-bearing mice and cancer patients is associated with impaired differentiation and accumulation of DCs (115–117). Unfortunately, the underlying mechanism(s) is not fully understood. In a murine model of allergic airway inflammation, LPS exposure promoted the development of a group of myeloid cells in the lung that resembled MDSCs phenotypically and functionally. These cells inhibited the reactivation of primed Th2 cells by DCs (118). In mice with hepatocellular carcinoma, MDSC-derived IL-10 was found to be responsible for the impaired TLR ligand-induced IL-12 production and T-cell stimulatory activity of DCs (116). Recently, it was shown that MDSC-mediated suppression of antigen presentation from DCs to CD4^+^ T cells depends on NO, which may cause nitration of STAT1, a key mediator for antigen presentation, and, consistently, this suppression is abrogated by iNOS inhibitors (11). In another recent study, Notch and STAT3 signals were found to be required by MDSCs to suppress the differentiation, maturation, and antigen presentation ability of DCs *in vitro* and *in vivo* (119).

Due to their superior antigen presentation and T-cell activation properties, DCs are utilized as cancer vaccines to prompt immunity against malignant cells. DC vaccines loaded with tumor antigens through various approaches aim to induce and potentiate tumor antigen-specific T-cell response. In line with MDSC-mediated suppression of DCs, favorable therapeutic efficacy of DC vaccination is associated with a reduced level of MDSCs in tumor-bearing mice (120, 121). In cancer patients, when monocyte-derived DCs are used as vaccines, the presence of CD14^+^HLA-DR^−/low^ MDSCs in the starting monocyte population causes impairment of DC maturation, antigen uptake, migration, and T-cell stimulation capacity (122). Therefore, it is reasonable to apply DC-based vaccines in combination with agents that target MDSCs. These agents include chemotherapeutics (e.g., all-trans retinoic acid, gemcitabine, and cyclophosphamide) (123, 124), tyrosine kinase inhibitors (e.g., sunitinib, axitinib, and dasatinib) (125–127), lenalidomide (128), and anti-Gr-1 antibody (120), and these combinations have shown reduced levels of MDSCs and improved efficacy in pre-clinical studies. The initiation of immune response by DC vaccines involves interaction between multiple immune cell types. Therefore, to overcome the immunosuppression mediated by MDSCs and maximize efficacy, further research is still needed to accurately define the action of MDSCs and other immune cells in DC vaccine-induced anti-tumor immunity.

### B Cells

In recent years, B cells have emerged as a novel target of MDSCs. In an *in vitro* model of B lymphopoiesis, MDSCs induced by adipocyte-derived factors inhibited B-cell development through IL-1 production (129). PMN-MDSCs inhibited the recruitment, proliferation, and cytokine secretion of B cells in the central nervous system of mice with experimental autoimmune encephalomyelitis (130). In the settings of retroviral infection and autoimmune disease, several animal studies have revealed that MDSCs impair B cell response by many of the mechanisms utilized in T-cell suppression, such as ROS, iNOS, ARG1, TGF-β, and PGE2 (54, 131). MDSCs from mice infected with retrovirus express V-domain Ig-containing suppressor of T-cell activation (VISTA), a negative checkpoint regulator that is homologous to PD-L1 and inhibits T-cell response, and VISTA deficiency in MDSCs or neutralization of VISTA by blocking antibody partially rescues the impaired B-cell proliferation (132). Both contact-dependent and contact-independent inhibition have been implicated in these studies (54, 131).

Whether these suppressive mechanisms are used by MDSCs in cancer settings is less well-elucidated. ROS, ARG1, iNOS PGE2, and TGF-β have recently been suggested to exert suppressive effects on B-cell proliferation and antibody production by tumor-induced MDSCs (133). In a murine model of lung cancer, the impeded B cell differentiation was associated with tumor progression and MDSC infiltration; mechanistically, MDSCs inhibit B cell response by TGF-β-mediated modulation of IL-7 and downstream STAT5 signaling, which are both essential in B-cell differentiation and function (133). In another study, Ku et al. showed that tumor-induced MDSCs reduce L-selectin on naïve CD4^+^ and CD8^+^ T cells and that even moderate L-selectin reduction is sufficient to profoundly disrupt homing of T cells to distant lymph nodes. Interestingly, the loss of L-selectin has also been found in B cells. In the study concerned, the shedding of L-selectin from naïve T cells and B cells was contact-dependent and was independent of major L-selectin sheddase ADAM17. Since the trafficking of both naïve B cells and CD4^+^ precursors of follicular helper T cells was hindered, the authors suggested that the T cell-dependent antibody production in lymph nodes may have been severely impaired (134).

Regulatory B cells (Bregs) are immunosuppressive and inhibit the expansion of pathogenic T cells and other pro-inflammatory lymphocytes through the production of IL-10, IL-35, and TGF-β. In consistence with these properties, Bregs have been shown to suppress anti-tumor immunity and promote tumor growth. In patients with colorectal cancer, the level of Bregs positively correlates with disease stage and with the frequency of MDSCs (135). In a murine model of breast cancer, Shen et al. showed that MDSCs upregulate PD-L1 expression on B cells and dampen their anti-tumor response; more interestingly, MDSCs may transform B cells into a novel subtype of Bregs that possesses higher inhibitory capability on T cells as compared with other subsets of Bregs (136). In another study, MDSCs induced the expansion of IL-10-producing Bregs, probably through iNOS, and ameliorated autoimmunity in mice with systemic lupus erythematosus (137). Conversely, in mice infected with retrovirus, M-MDSCs inhibited the proliferation of IL-10-producing Bregs in response to LPS stimulation (54).

## Antigen-Specific and Non-specific Suppression of CD8^+^ and CD4^+^ T Cells

**Figure 2:**
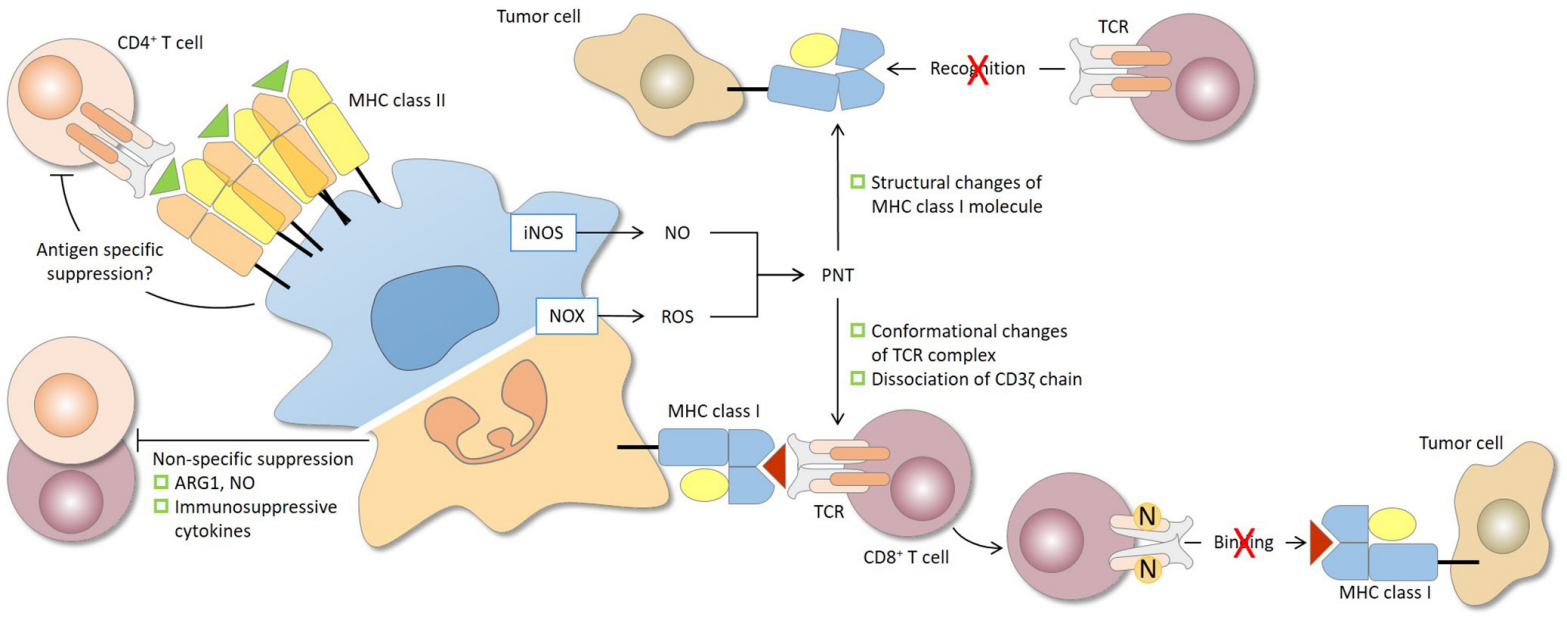
Antigen specificity of MDSC-mediated suppression of CD4^+^ and CD8^+^ T cells. The antigen specificity of T-cell suppression by MDSCs is determined largely by the characteristics of the effector molecules involved. The short-lived reactive oxygen species (ROS) and peroxynitrite (PNT) are responsible for antigen-specific suppression, provided that MDSCs and T cells are in close contact, while arginase 1 (ARG1), nitric oxide (NO), and immunosuppressive cytokines, which have relatively longer half-lives, mediate antigen-non-specific suppression. During the close and prolonged interaction between MDSCs and CD8^+^ T cells in antigen recognition, PNT causes nitration and conformational changes of the TCR complex and dissociation of CD3ζ molecules. CD8^+^ T cells consequently lose their binding ability to peptide-MHC class I complex and are rendered non-responsive to specific peptide presented by tumor cells. PNT may also induce structural changes of MHC class I on tumor cells, leading to reduced antigenic peptide binding. In this case, antigen-specific CD8^+^ T cells, even if functional, fail to recognize tumor cells. For CD4^+^ T cells, antigen-specific suppression by MDSCs has been reported and may require sufficient MHC class II expression by MDSCs. iNOS, nitric oxide synthase; NOX2, NADPH oxidase 2.

Among the various cell targets, suppression of T cells remains the characteristic necessary to define bona fide MDSCs, provided that the phenotypic criteria are met. With the abovementioned mechanisms, MDSCs are capable of suppressing both antigen-specific and non-specific T-cell response (Figure 2). It is now generally accepted that ROS, and PNT in particular, are responsible for antigen-specific suppression, provided that MDSCs and T cells are in close contact, since these substances are unstable and short-lived, while iNOS, ARG1, and immunosuppressive cytokines are responsible for antigen-non-specific suppression, since effector molecules of these mechanisms have relatively longer half-lives and require cellular proximity, but not close interaction, to exert inhibition (1).

Early studies have shown that Gr-1^+^ immature myeloid cells isolated from tumor-bearing mice are able to uptake and process soluble proteins and present the antigenic epitopes on their surface (97). Their suppression of antigen-specific CD8^+^ T cells requires antigen presentation via MHC class I and ROS production (14, 138). Studies in the last decade have revealed that MDSC-induced antigen-specific T-cell tolerance results from post-translational modification of the TCR complex. MDSCs from gp91^phox−/−^ mice produce little ROS and fail to inhibit CD8^+^ T cells, and neutralization of PNT abrogates the suppressive activity of MDSCs on T cells (17). Nagaraj et al. demonstrated that the close and prolonged cell-to-cell contact during antigen recognition allows MDSC-derived PNT to cause nitration of tyrosines in the TCR-CD8 complex, which induces conformational changes in these molecules and leads to loss of binding ability to peptide-MHC complex (17). Consistently, using double TCR transgenic CD8^+^ T cells, the same group later showed that MDSCs induce CD8^+^ T-cell tolerance only against the peptide presented by themselves, while they do not affect T-cell response to peptide specific for other TCR that is not presented by MDSCs (139). In accordance with previous findings, the authors showed that nitration of surface molecules of T cells is localized to the site of physical interaction between MDSCs and T cells, which may lead to dissociation between TCR and CD3ζ molecules, and consequently, nitrotyrosine positive CD8^+^ T cells are rendered non-responsive to specific peptide (139). In another study, however, ROS were found not to be involved in antigen-specific T-cell suppression by MDSCs, and MDSCs deficient in MHC class I showed no impairment in antigen-specific suppression, which excludes the necessity of antigen presentation (9).

Interestingly, PNT produced by MDSCs can facilitate immune evasion of tumor cells even in the presence of normal functioning T cells. PNT induces nitration and structural changes of MHC class I molecules on tumor cells, which hampers their capacity to bind antigenic peptide and subsequently impairs the recognition by CTLs, therefore affording tumor cells resistance to antigen-specific CTLs (140). These findings collectively suggest the involvement of multiple mechanisms in antigen-specific CD8^+^ T-cell suppression by MDSCs.

On the other hand, evidence for MDSC-mediated antigen-specific suppression of CD4^+^ T cells remains elusive, and different results have been reported. It was previously indicated that MDSCs fail to suppress antigen-specific CD4^+^ T-cell proliferation, which may be due to the low MHC class II expression on MDSCs, which precludes them from forming close contact with CD4^+^ T cells (91, 138). However, MDSC-mediated suppression of the proliferation of CD4^+^ T cells exposed to a specific peptide has been reported, which is at least partially due to cysteine deprivation by MDSCs (25, 88). Interestingly, Nagaraj and colleagues have shown that MDSCs are able to suppress antigen-specific CD4^+^ T-cell response *in vitro* and *in vivo*, as long as their MHC class II expression reaches a sufficient level (141). In different experimental systems, MDSCs are able to blunt IFN-γ production of both tumor-specific CD8^+^ and CD4^+^ T cells in the spleen of tumor-bearing mice *in vivo* (142). In patients with liver cancer, depletion of CD14^+^HLA-DR^−/low^ M-MDSCs enhances IFN-γ secreting CD4^+^ T cells specific to α-fetoprotein (57). These discrepancies might be explained, in part, by the varied MHC class II level of MDSCs that has been described in different tumor models and human studies, and under some experimental conditions, MDSCs could inhibit the proliferation of T cells without affecting the IFN-γ production and vice versa (3, 4).

## Regulation on the Suppressive Nature of MDSCs

**Figure 3:**
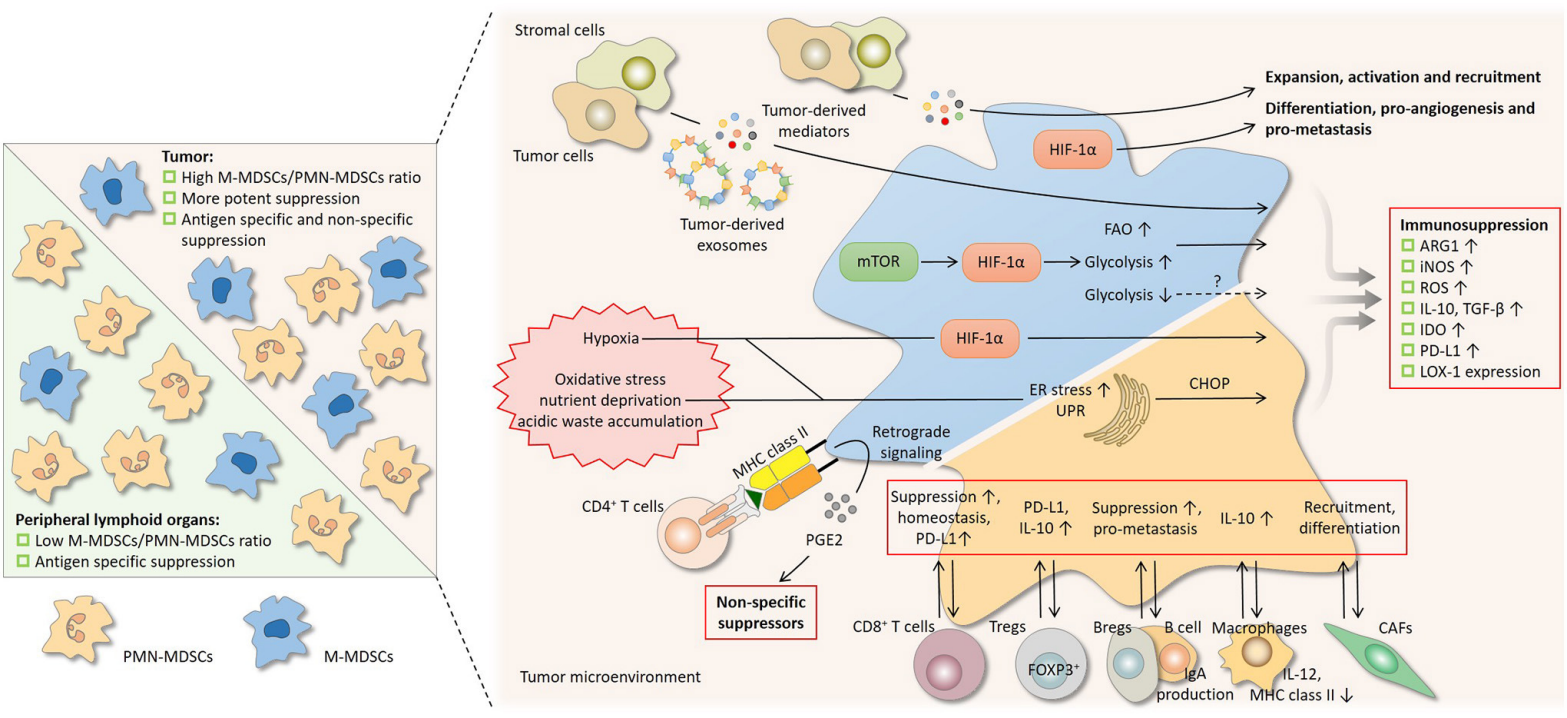
Regulation on the suppressive nature of MDSCs by subset composition and the tumor microenvironment. Differential suppressive capacity and mechanism(s) between PMN-MDSCs and M-MDSCs influence the suppression by MDSCs as a whole population (left). In peripheral lymphoid organs where PMN-MDSCs predominate, suppression by MDSCs is mainly antigen-specific, since the activity of PMN-MDSCs depends largely on reactive oxygen species (ROS) and peroxynitrite (PNT). In tumor where the proportion of M-MDSCs increases, suppression is more potent and is both antigen-specific and non-specific, since M-MDSCs are more suppressive and mainly rely on arginase 1 (ARG1), nitric oxide (NO), and immunosuppressive cytokines. A network of cytokines, hostile physical conditions, and cells in the tumor microenvironment regulates MDSCs in multiple aspects (right). Soluble mediators derived from tumor regulate the suppressive activity of MDSCs and also drive their development. After being taken by MDSCs, the contents of tumor-derived exosomes, which act as intercellular messengers, promote the expansion and potentiate the suppressive capacity of MDSCs. Like tumor cells, MDSCs undergo metabolic reprogramming to adapt to varying surroundings. Hypoxia-inducible factor 1α (HIF-1α) induced by the mammalian target of rapamycin (mTOR) pathway enhances glycolysis and may potentiate suppression by MDSCs, whereas glycolysis has also been reported to be a negative regulator. The heightened fatty acid oxidation (FAO) is associated with upregulated ARG1 and increased NO and PNT production. Hypoxic signaling, primarily through HIF-1α, is another central regulator. HIF-1α promotes many non-immunological activities of MDSCs, including differentiation, pro-angiogenesis, and pro-metastasis. HIF-1α augments MDSC-mediated suppression by upregulating several effector molecules. The hostile conditions in the tumor milieu, such as oxidative stress, nutrient deprivation, and acidic waste accumulation, causes ER stress and induce unfolded protein response (UPR) in MDSCs. ER stress response marker C/EBP homologous protein (CHOP) regulates ARG1, superoxide, and PNT production by MDSCs. The bidirectional communication with stromal cells fine-tunes the induction, homeostasis, differentiation, and suppressive function of MDSCs. Bregs, regulator B cells; CAFs, cancer-associated fibroblasts; DCs, dendritic cells; iNOS, nitric oxide synthase; LOX-1, lectin-type oxidized LDL receptor-1; NK, natural killer; PD-L1, programmed cell death-ligand 1; Tregs, regulatory T cells.

In most studies, immunosuppressive activity is detected only in MDSCs derived from tumor-bearing host but not in their control counterparts from tumor-free host, suggesting a tight control over MDSCs by tumor. MDSCs carry out immune suppression principally in the tumor microenvironment, which is a highly dynamic complex and plays a crucial role in tumor development. The constant bi-directional communication between MDSCs and the ever-changing microenvironment shapes the phenotype and function of MDSCs (Figure 3). For instance, tumor-derived M-MDSCs show higher suppression of T cells than spleen- or bone marrow-derived M-MDSCs from the same mice. Several cellular and non-cellular components of the tumor microenvironment, including the subset composition of MDSCs, tumor cells, stromal cells, cytokines, metabolic state, and hypoxia, regulate the suppressive nature of MDSCs.

### Subset Composition and Antigen Specificity and Capacity of MDSC-Mediated Suppression

It is now clear that the suppressive machineries of MDSCs do not act simultaneously, and subsets of MDSCs use different mechanisms for T-cell suppression (9, 10, 92, 143). For instance, M-MDSCs, whose activity mainly relies on ARG1, NO, and immunosuppressive cytokines, inhibit both antigen-specific and non-specific T-cell response (8, 10, 19, 92, 143, 144), while PMN-MDSCs, whose activity largely depends on high ROS and PNT production, inhibit T cells in an antigen-specific manner (10, 92). In one study, only M-MDSCs, but not PMN-MDSCs, were able to augment the activation-induced Fas upregulation of CD8^+^ T cells through NO production and sensitize them to Fas-mediated apoptosis and were able to impede the differentiation of mature CTLs (143). Therefore, the suppressive nature of MDSCs is influenced by their subset composition.

PMN-MDSCs is commonly the predominant subpopulation in peripheral lymphoid organs in many murine tumor models, and accordingly, antigen-specific T-cell tolerance is detected at these sites (145, 146). This peripheral antigen-dependent T-cell inhibition may partially explain the findings in some studies that T cells in the periphery retain their responsiveness to other non-specific stimuli (3, 17, 97). On the other hand, the proportion of M-MDSCs is substantially higher in the tumor milieu (144, 145), and in spite of the common findings that PMN-MDSCs may still be the prevalent subpopulation, M-MDSCs are more suppressive than PMN-MDSCs on a per-cell basis (9, 119). As a consequence, tumor-infiltrating MDSCs demonstrate higher immunosuppressive capacity than their peripheral counterparts and are able to inhibit both antigen-specific and non-specific T-cell function (19, 147, 148).

In spite of these findings, it is noteworthy to point out that similar or even stronger inhibitory capacity of peripheral MDSCs has also been reported (140, 149, 150) and that non-specific T-cell suppression is not uncommon in MDSCs derived from peripheral lymphoid organs (149, 151).

It is common that the ratio between subgroups of MDSCs varies in different tumor models. Unfortunately, many of these studies have not addressed the subset composition of intratumoral or peripheral MDSCs in detail, nor have they assessed the suppressive capacity of PMN-MDSCs and M-MDSCs separately. Therefore, the discrepancies on antigen specificity and capacity of MDSC-mediated suppression, on the one hand, should be interpreted with care, and on the other hand, may suggest that subset composition of MDSCs is not likely the sole nor a major determinant that influences their suppressive nature.

### Tumor-Derived Mediators

The generation of MDSCs includes two phases. Firstly, aberrant myelopoiesis and blocked differentiation of immature myeloid cells lead to the expansion of MDSCs, mainly driven by various growth factors; secondly, these MDSCs are activated to be fully functional, primarily promoted by pro-inflammatory factors. This two-signal model of expansion and activation may answer the question of why MDSCs are not generated under normal physiological settings or during acute inflammation. In steady state, growth factors stimulate normal hematopoiesis without generating MDSCs due to the absence of pro-inflammatory factors, whereas during acute inflammation, in the absence of sustained growth factors, pro-inflammatory factors alone do not lead to MDSC generation either, since immature myeloid cells may rapidly differentiate into mature myeloid cells.

As discussed in the previous sections, many of the tumor-derived mediators actively regulate the suppressive function of MDSCs. In a murine model of tissue-specific inflammatory response, MDSCs from inflammatory or tumor site are more suppressive than MDSCs from spleen, and splenic MDSCs from inflamed mice are more suppressive than splenic MDSCs from naïve mice (148). Further study from the same group has shown that MDSCs exposed to IFN-γ, IL-13, and GM-CSF *in vitro* or MDSCs localized in inflammatory or tumor site *in vivo* have elevated L-arginine transporter cationic amino acid transporter 2 expression, which parallels the expression of ARG1 and iNOS and is required for optimal suppressive activity of MDSCs (146). These findings suggest a priming effect of tumor-derived pro-inflammatory cytokines. In a more recent study, tumor cells upregulate tumor necrosis factor-α-induced protein 8-like 2 (TIPE2) in MDSCs through ROS, which in turn controls the polarization of MDSCs by increasing pro-tumoral and inhibiting anti-tumoral mediator expression (152).

Several pro-inflammatory factors are reported to enhance the suppressive potency of MDSCs. For instance, PGE2 generated by COX2 in tumor cells upregulates ARG1 expression of MDSCs through the EP4 receptor (153). PGE2 promotes hypermethylation and repression of a cluster of myeloid genes, which is in contrast to the profile from DCs generated *in vitro* or CD11b^+^ cells from healthy controls. This MDSC-specific gain of methylation requires the upregulation of DNA methyltransferase 3A, while its downregulation abolishes the immunosuppressive properties of MDSCs (154). It another study, PGE2 potentiates the suppressive function of human M-MDSCs induced by GM-CSF/IL-6 from peripheral blood mononuclear cells (155). However, whether these actions of PGE2 occur *in vivo* remains to be determined. IL-17 not only enhances tumor-infiltrating MDSCs, probably by increasing CXCL1 and CXCL5 secretion by tumor cells, but also potentiates their inhibition on T cells through upregulation of ARG1 and IDO (156). The calcium-binding pro-inflammatory proteins S100A8 and S100A9, which are ubiquitously present in the tumor microenvironment, drive the accumulation of MDSCs through increased recruitment to primary tumor and pre-metastatic niche (150). It was recently reported that S100A8 enhances T-cell suppression by MDSCs (157) and that S100A9 induces IL-6 and IL-10 release by MDSCs (158). Furthermore, MDSCs also express and secret S100A8/A9, thus forming a positive feedback loop that helps to maintain suppressive MDSCs in the tumor microenvironment (150).

Both type I and II interferons upregulate PD-L1 expression in MDSCs. It is well-documented that IFN-γ functions as a master regulator of PD-L1 expression in tumor. IFN-γ neutralization reduces tumor-infiltrating PD-L1^+^ MDSCs *in vivo*, and mechanistically, IFN-γ upregulates IRF1, which in turn binds to IRF-binding sequence in *cd274* promoter and activates PD-L1 expression (72). The IFN-γ level in the tumor microenvironment may be reduced due to MDSC-mediated suppression of T cells and NK cells, which are important sources of IFN-γ. As a compensatory mechanism, MDSCs may maintain their PD-L1 expression by secreting IFN-α and IFN-β, which bind to IFN receptor type I and upregulate PD-L1 in an autocrine manner (159).

It is noteworthy to point out that many of the tumor-derived mediators influence more than one aspect of MDSCs. For instance, in addition to promoting expansion, GM-CSF alone is able to promote immunosuppression by MDSCs (160). GM-CSF increases IL-4Rα expression on MDSCs, which leads to IL-13-induced ARG1 upregulation (161), and GM-CSF drives PD-L1 and IDO expression of MDSCs through STAT3 activation (69, 80). Tumor-derived migration inhibitory factor has been reported to promote the differentiation, recruitment, and suppressive activity of MDSCs (162, 163). These pleiotropic and redundant effects further complicate the regulatory network of MDSC development.

### Tumor-Derived Exosomes

Exosomes are small extracellular vesicles released by nearly all cells and are present in most body fluids. These membrane-bound vesicles contain proteins, DNA, mRNA, and miRNA and act as intercellular messengers (164). Tumor constantly produces and secrets exosomes. Upon contact with target cells, tumor derived-exosomes are able to alter the phenotypic and functional characters of the recipients, reprogramming them into participants in tumor progression. In the early phase of tumor growth, exosomes derived from immune cells in the tumor microenvironment may facilitate anti-tumor response, while in more advanced disease, tumor derived-exosomes promote immune suppression by interfering with the differentiation, maturation, and anti-tumor activity of immune cells (164). Several recent studies have shown that MDSCs also produce exosomes, whose contents are implicated in their own chemotaxis, survival, pro-metastatic, and immunosuppressive activity (165).

Studies have shown that tumor-derived exosomes promote the expansion of MDSCs. Administration of tumor-derived exosomes to healthy mice leads to increased frequency of immature myeloid cells that acquire the phenotypic and functional characters of MDSCs (166). Tumor derived-exosomes induce accumulation of splenic and intratumoral MDSCs that are able to promote tumor growth, which is dependent on exosomal PGE2 and TGF-β (167). In multiple myeloma, exosomes derived from both tumor cells and stromal cells expand MDSCs (168, 169). In addition, tumor derived-exosomes may contribute to metastasis by inducing accumulation of MDSCs, PMN-MDSCs in particular, in the pre-metastatic niche (170, 171).

Many of the suppressive machineries of MDSCs can be potentiated by tumor derived-exosomes, including expression of ARG1 and iNOS, and production of IL-10 and VEGF (48, 167, 169, 172). The suppressive capacity of MDSCs on T cells is accordingly heightened (48, 169). STAT3 is implicated in this exosomal regulation on MDSCs (169). Chalmin et al. have shown that HSP72 expressed on tumor derived-exosomes induces suppressive activity of MDSCs, which activates STAT3 in a TLR2/MyD88-dependent manner through autocrine production of IL-6 (142). Similarly, in another study, MDSCs were expanded and activated by exosomal HSP70, which induced phosphorylation of STAT3 through the TLR2/MyD88 pathway (172). In consistence with these findings, T-cell proliferation is inhibited by MDSCs isolated from mice treated with tumor derived-exosomes but not by MDSCs isolated from MyD88 knockout mice treated with tumor derived-exosomes (171). Furthermore, stromal cell-derived exosomes are also reported to enhance T-cell suppression by MDSCs, probably through the STAT3 pathway as well (168).

Tumor-derived exosomes are able to mediate RNA transfer from tumor cells to recipient cells. Ridder and colleagues have shown that MDSCs are the major recombined cells in the tumor microenvironment after the uptake of exosomes and their RNA content and that MDSCs recombined with exosomal RNA display enhanced ARG1, TGF-β, and PD-L1 expression as compared to the non-recombined counterparts (173). In a recent study, hypoxia increases exosome secretion by glioma cells. Moreover, hypoxia upregulates miR-10a and miR-21 in glioma-derived exosomes, which in turn potentiates the suppressive function of MDSCs (174).

### Metabolic Reprogramming of MDSCs

Along with disease progression, malignant cells undergo dramatic alteration in their energy metabolism to meet the demand for rapid tumor growth and to adapt to the varying microenvironment. Meanwhile, it was recently demonstrated that tumor-associated immune cells also experience metabolic changes that help to shape their pro- and/or anti-tumor response (175). In this regard, metabolic reprogramming is emerging as a regulator of MDSCs. Using MSC-1 cells, an immortalized murine MDSC cell line, early *in vitro* studies have revealed two distinct bioenergetic states that coincide with the exponential and stationary growth phases of MSC-1 cells (176) and that their maturation and suppressive potential are accompanied by an increase in the central carbon metabolism activity (177).

MDSCs exhibit a high glycolytic rate (175). The enhanced glycolysis of MDSCs helps to keep their ROS level within a safe range and promotes their survival and accumulation in tumor-bearing host (178). mTOR-mediated HIF-1α induction is essential in glycolytic activation (175). Inhibiting the mTOR pathway blocks the differentiation of M-MDSCs from precursors by impairing glycolysis. Consistently, 2-deoxyglucose, which inhibits glycolysis, blocks the differentiation of M-MDSCs, while metformin, which promotes glycolysis, rescues the reduction in M-MDSCs caused by mTOR inhibition (179). On the other hand, glycolysis in tumor cells also contributes to the expansion of MDSCs, which is mediated by increased production of G-CSF, GM-CSF, and lactate (100, 180).

In addition to promoting expansion, glycolysis regulates the function of MDSCs. A recent study has found that enhanced glycolysis mediated by the mTOR pathway leads to stronger suppressive capacity of tumor-infiltrating M-MDSCs as compared with splenic M-MDSCs and that mTOR inhibition by rapamycin reduces the glycolysis, intratumoral level, and suppressive activity of M-MDSCs (181). Attenuated iNOS and ARG1 may be responsible for the impaired function caused by rapamycin-mediated glycolysis inhibition (179).

On the contrary, glycolysis as a negative regulator of MDSCs has also been reported. It is shown that mTOR- and HIF-1α-induced glycolytic activation is required for differentiation of MDSCs to a less suppressive M1 phenotype (182). In the settings of transplantation and autoimmune disorder, dexamethasone expands MDSCs and strengthens their function. In a model of immunological hepatic injury, dexamethasone inhibits HIF-1α-dependent glycolysis in MDSCs and promotes their suppressive activity to protect against inflammatory injury (183). In addition, there are studies showing that mTOR inhibition by rapamycin potentiates the suppressive activity of MDSCs, which protects against acute graft-versus-host disease and acute kidney injury (184, 185); yet, unfortunately, the glycolytic or other metabolic characteristics of MDSCs were not determined in these studies. These seemingly conflicting results indicate the complexity and the possibly context-dependent manner in which glycolytic rate determines the function of MDSCs.

Recently, it is shown that tumor-infiltrating MDSCs have increased fatty acid oxidation (FAO), which is accompanied by upregulated ARG1, increased NO, and PNT production, and that FAO inhibition impairs the suppressive activity of MDSCs *in vitro* and *in vivo* (186). Only intratumoral MDSCs, and not splenic MDSCs, have increased FAO, suggesting that the microenvironment is responsible for this metabolic alteration (186). Consistently, a further study from the same group demonstrated that tumor-derived cytokines, such as G-CSF and GM-CSF, induce the expression of lipid transport receptors in intratumoral MDSCs through the activation of STAT3 and STAT5, which leads to increased uptake of lipids that are present at high concentrations in the tumor microenvironment; intracellular accumulation of lipids in turn increases the oxidative metabolism and suppressive activity of MDSCs (187).

### Hypoxia and HIF-1α

Hypoxia caused by excessive oxygen consumption by tumor cells and aberrant organization of tumor vasculature is a common feature of the tumor microenvironment and plays a central role in tumor progression, primarily through HIF-dependent signalings. Multiple activities of MDSCs are regulated by hypoxia. For instance, hypoxia facilitates the recruitment of MDSCs to tumor site (188, 189). Intratumoral MDSCs preferentially localize in poorly perfused and hypoxic regions, and their pro-angiogenic capacity is generally enhanced by hypoxia (6). The homeostasis of tumor-infiltrating MDSCs is fine-tuned by the hypoxic microenvironment, since hypoxia promotes the differentiation of intratumoral MDSCs to TAMs (190), while it is also reported that hypoxia promotes the maintenance of MDSCs by upregulating ectonucleoside triphosphate diphosphohydrolas 2 in tumor cells, which forms a 5′-AMP-rich microenvironment and prevents differentiation of MDSCs (191).

It is now generally accepted that microenvironmental hypoxia directly augments the suppressive function of MDSCs (1). In a tumor model with similar PMN-MDSC to M-MDSC ratios in spleen and tumor site, Corzo et al. found that the inhibition on T cells is antigen-specific by splenic MDSCs, which display higher ROS production, while it is both antigen-specific and non-specific by tumor-infiltrating MDSCs, which exhibit upregulated ARG1 and iNOS. Exposure of splenic MDSCs to hypoxia leads to non-specific T-cell suppression, suggesting that the hypoxic microenvironment may convert MDSCs into non-specific suppressors. This conversion is mediated by HIF-1α (190). A similar difference in suppressive mechanisms and antigen specificity is detected in MDSCs obtained from peripheral blood and tumor tissue of patients with head and neck cancer (190). Similarly, it was recently reported that HIF-1α potentiates the immunosuppressive activity of splenic MDSCs in a murine model of chronic *Leishmania* infection (192).

Noman et al. have shown that the PD-L1 level is higher on intratumoral MDSCs than on splenic MDSCs and that hypoxic stress upregulates PD-L1 on splenic MDSCs through HIF-1α. More importantly, hypoxia potentiates the ability of splenic MDSCs to suppress both specific and non-specific stimuli-mediated T-cell proliferation, while PD-L1 blockade abrogates the enhanced suppression under hypoxia, in part by decreasing the production of suppressive cytokines, particularly IL-6 and IL-10, in hypoxic MDSCs (43). The authors have also found that hypoxia increases the secretion of IL-6, IL10, and TGF-β from MDSCs (43). In another study from the same group, tumor-infiltrating MDSCs expressed an increased level of miR-210 as compared with splenic MDSCs, and hypoxia induced miR-210 in splenic MDSCs via HIF-1α. MiR-210 in turn enhanced the suppressive capacity of splenic MDSCs by increasing their ARG1 activity and NO production without affecting ROS, IL-6, or IL-10 production or PD-L1 expression (67). In a more recent study, HIF-1α acted as a transcriptional activator of VISTA, a negative checkpoint regulator in the B7 family, in MDSCs and consistently, antibody blockade or genetic ablation of VISTA abolished MDSC-mediated suppression of T cells under hypoxic but not normoxic conditions (193). These findings suggest that hypoxia regulates MDSC-mediated suppression through multiple pathways.

MDSCs actively participate in tumor metastasis by inducing EMT, increasing the invasiveness and stemness of tumor cells, and stimulating angiogenesis (5, 6). Unfortunately, the precise roles of hypoxia and hypoxic signalings in these MDSC-driven steps of metastatic cascade are not well-defined. On the other hand, MDSCs actively participate in pre-metastatic niche formation. MDSCs, especially the granulocytic subset, reach the pre-metastatic site prior to the arrival of disseminated tumor cells, which is regulated indirectly by hypoxia in the primary tumor. In a murine mammary tumor model, tumor that grows in pre-irradiated mammary tissue has decreased vascular density and is more hypoxic and metastatic, recapitulating the clinical features of locally relapsed breast cancer after radiation therapy; HIF-1-dependent Kit ligand expression by hypoxic tumor cells mobilizes c-Kit^+^ PMN-MDSCs to home to pre-metastatic lungs to promote metastasis (194). In other studies, PMN-MDSCs are recruited by MCP-1 or G-CSF derived from hypoxic tumor cells to pre-metastatic lungs, where they may inhibit the cytotoxicity of NK cells (195, 196).

### Endoplasmic Reticulum Stress and Unfolded Protein Response

In homeostatic settings, the endoplasmic reticulum (ER) readily handles the folding of secretory and transmembrane proteins. The hostile conditions in the tumor milieu, such as hypoxia, oxidative stress, nutrient deprivation, and acidic waste accumulation, impair the protein-folding capacity of ER, thus provoking a cellular state of ER stress. When the misfolded proteins exceed a tolerable level, PKR-like ER-resident kinase (PERK), inositol-requiring enzyme 1α (IRE1α), and activating transcription factor 6α (ATF6α) detect the presence of ER stress and trigger unfolded protein response (UPR) to improve the folding efficiency in ER (197). These ER-localized sensors are held inactive by chaperone BiP in steady state, while upon ER stress, the dissociation of BiP activates all three sensors: PERK phosphorylates the translation initiation factor eIF2α, which restricts cap-dependent translation and in turn upregulates activating transcription factor 4 (ATF4) and its downstream target C/EBP homologous protein (CHOP); IRE1α cleaves the X-box-binding protein 1 (XBP1) mRNA, and the spliced mRNA is re-ligated to produce highly active XBP1s that regulates gene expression involved in protein folding; ATF6α fine-tunes UPR by regulating the transcription of ER chaperone genes (197).

Unresolvable ER stress often leads to cell death, while tolerable defect in protein-folding capacity may fuel tumor cell survival, metastasis, angiogenesis, and therapeutic resistance. The immunosuppressive effect of ER stress is receiving growing attention (197). Mahadevan et al. have shown that stressed tumor cells actively regulate the function of myeloid cells. For instance, tumor cells undergoing ER stress release yet unidentified soluble mediators that lead to upregulated UPR markers and pro-inflammatory cytokines in responder macrophages (198). This transmissible ER stress also imprints bone marrow-derived DCs with increased ARG1 and decreased ability to cross-present antigen to CD8^+^ T cells (199). On the other hand, intrinsic ER stress regulates the myeloid cell activity as well. STAT3 synergizes with STAT6 in macrophages to promote cathepsin secretion and tumor invasion through the IRE1α pathway (200). ER stress and XBP1 activation in tumor-infiltrating DCs lead to abnormal lipid accumulation, which impairs their antigen presentation capacity (201).

In line with macrophages and DCs, MDSCs exhibit clear signs of ER stress and UPR. MDSCs isolated from tumor-bearing host have a higher level of ER stress response markers as compared with monocytes and neutrophils from the same host or healthy control (202). Furthermore, the CHOP level in tumor-infiltrating MDSCs is higher than in splenic MDSCs or other tumor-infiltrating immune cells (203).

Recent studies have demonstrated that ER stress response regulates the homeostasis and suppressive function of MDSCs. ER stress induces apoptosis of MDSCs through upregulation of TRAIL-R or through the eIF2α-ATF4-CHOP pathway; though the lifespan of MDSCs is shortened by ER stress, it may stimulate myelopoiesis and the turnover of MDSCs in tumor-bearing host (202, 203). Administration of the ER stress inducer thapsigargin promotes infiltration of MDSCs in tumor and enhances their suppressive capacity through upregulation of ARG1, iNOS, and NOX2 (204).

Thevenot et al. have elaborately shown that the suppressive activity of MDSCs is regulated by ER stress response marker CHOP (203). In CHOP-deficient mice, tumor growth is significantly retarded, while it is partially restored by depletion of MDSCs, suggesting a reversal of the tumor-promoting activity of MDSCs. Consistently, functional assessment of tumor-infiltrating CHOP^−/−^ MDSCs reveals reduced suppression of T cells, which is associated with decreased ARG1, superoxide, and PNT; furthermore, these CHOP^−/−^ MDSCs acquire a DC-like phenotype and are able to stimulate immune response.

In another study, ER stress-related genes were found to be among the most upregulated in PMN-MDSCs, as compared with neutrophils from the same cancer patient or a healthy individual (205). Surface expression of lectin-type oxidized LDL receptor-1 (LOX-1), which is regulated by ER stress, effectively distinguishes immunosuppressive PMN-MDSCs from neutrophils in cancer patients (205). ER stress induced by thapsigargin promotes LOX-1 upregulation in human neutrophils and converts them into immunosuppressive cells, which is prevented by inhibiting the IRE1α-XBP1s pathway (205, 206). However, whether downstream signaling through LOX-1 is responsible for the acquisition of suppressive activity by neutrophils remains undetermined.

### Crosstalk Between MDSCs and Stromal Cells in the Tumor Microenvironment

Many of the tumor-promoting roles of MDSCs, such as immune suppression, pro-angiogenesis, and pro-metastasis, are regulated by the surrounding cells in the tumor microenvironment. How tumor cells regulate the immunosuppressive function of MDSCs has been discussed in previous sections. The induction and suppressive capacity of MDSCs are also fine-tuned during the dynamic and mutualistic communication with the non-malignant stromal cells in the tumor milieu. Many of these cells are not merely targets but also regulators of MDSCs.

MDSCs primarily inhibit T-cell response, and on the other way round, T cells influence the suppressive nature of MDSCs. The antigen-specific CD4^+^ T cells, and not CD8^+^ T cells, enhance the immunosuppressive capacity of MDSCs by turning them into non-specific suppressors *in vitro* and *in vivo*. Mechanistically, this effect requires cross-linking of MHC class II on MDSCs during cell-to-cell contact with activated CD4^+^ T cells, and the subsequent retrograde signaling in MDSCs upregulates COX2 and PGE2 expression (141). In a recent study, IFN-γ produced by T cells was found to be critical in regulating the enhanced suppressive activity of MDSCs induced by TLR2 ligand, which promoted differentiation of MDSCs into iNOS^+^ macrophages (207).

In addition to immunosuppression, T cells regulate the induction of MDSCs. It has been reported that FasL^+^-activated T cells may regulate the homeostasis of MDSCs through Fas-FasL interaction, which induces apoptosis of MDSCs (208). In human colorectal cancer, γδT cells promote the recruitment, proliferation, and survival of PMN-MDSCs through secretion of large amounts of IL-17 and other cytokines, including IL-8, GM-CSF, and TNF-α (209). It has been shown in different murine tumor models that TNF-α secreted by CD4^+^ T cells, and partially by CD8^+^ T cells, induces myelopoiesis, which increases the frequency of MDSCs (210).

PD-L1 expression on MDSCs is upregulated upon co-culture with T cells (79), and MDSCs are able to induce PD-1 expression on T cells through TGF-β (75, 211). In melanoma-bearing mice receiving IL-2- and TNF-α-coding adenovirus in combination with adoptive T-cell therapy, PD-L1 was upregulated in intratumoral MDSCs, and the frequency of PD-1^+^ CD8^+^ T cells correlated with the PD-L1 expression level on MDSCs in tumor site (70).

Not only do MDSCs promote Treg induction and recruitment: their suppressive function is also modified by Tregs. An earlier study reported that CD80 expression is required for MDSC-mediated antigen-specific T-cell suppression, which is dependent on CD4^+^CD25^+^ Tregs and CTLA-4 and that depletion of CD4^+^CD25^+^ Tregs diminishes the suppression mediated by MDSCs (212). In a more recent study, Treg depletion decreased PD-L1 expression and IL-10 production by MDSCs (73). In a murine model of melanoma, the expansion, recruitment, and activation of MDSCs occurred in a Treg-dependent manner and required the expression of IDO (213). Therefore, it is likely that MDSCs and Tregs do not act separately but rather cooperate reciprocally in immune suppression.

Crosstalk between MDSCs and B cells has been found recently. In one study, MDSCs that accumulated around the germinal center co-localized with B cells in the spleen of tumor-bearing mice, and cell-to-cell interaction through TNFR2 on MDSCs and membranous TNF on B cells promoted the proliferation and differentiation of B cells into IgA-producing plasma cells (214). Both IL-10 and TGF-β are crucial for this MDSC-mediated IgA response. In another study, Bregs from tumor-bearing mice increased the immunosuppressive and pro-metastatic function of MDSCs, partially through the TGF-β type I/II receptor signaling axis (215).

IL-10 is implicated in the interaction between MDSCs and other immune cells. Through cell-to-cell contact, MDSCs produce IL-10 to downregulate IL-12 by macrophages, and macrophages in turn stimulate IL-10 upregulation by MDSCs (216). The increased IL-10 level and reduced IL-12 level consequently skew the immunity toward a tumor-promoting type 2 response. In another recent study, MDSC-derived IL-10 decreased IL-6 and TNF-α while increasing NO produced by macrophages (217). Meanwhile, IL-10 produced by MDSCs may reduce MHC class II molecule expression on macrophages, leading to diminished antigen-presentation capacity (218). This bi-directional crosstalk between MDSCs and macrophages is accentuated by the inflammatory microenvironment. MDSCs isolated from tumor with a heightened IL-1β level produce more IL-10 and downregulate IL-12 by macrophages to a greater degree as compared with MDSCs from less inflammatory tumors (38). This IL-10 elevation by MDSCs requires IL-6 from macrophages and signaling through TLR4 on MDSCs and macrophages (38, 218). This action of inflammation is further corroborated by the findings that pro-inflammatory mediators PGE2 and HMGB1 upregulate IL-10 in MDSCs in the presence of macrophages (21, 218).

It is reported that mast cells not only induce the recruitment but also promote the suppressive function of MDSCs, probably through CD40L-CD40 interaction (219, 220).

Cancer-associated fibroblasts (CAFs) are a heterogeneous group of activated fibroblasts that play pleiotropic roles in tumor development and are able to modulate anti-tumor immunity on various levels. Through secretion of CCL2 and CXCL12, CAFs facilitate the recruitment of MDSCs (221). Meanwhile, pancreatic CAFs produce multiple MDSC-promoting soluble mediators, IL-6 in particular, and favor the differentiation of MDSCs (222). CAFs from hepatic cancer attract monocytes to the tumor microenvironment by CXCL12 and induce their differentiation into MDSCs through IL-6-mediated STAT3 activation (223). The MDSC-promoting effect of CAFs in breast cancer involves epigenetic regulation by histone deacetylase 6 (224). Consistently, inhibiting CAFs leads to reduced *in vivo* induction and intratumoral level of MDSCs (225, 226). On the other hand, MDSCs promote activation and migration of CAFs, suggesting a positive feedback loop that amplifies interaction between them. To further complicate the issue, in recent studies, CAFs show similar phenotypic and immunosuppressive characteristics to the circulating fibrocytes that may arise from MDSCs and may represent a novel MDSC subset (227).

## Conclusions and Perspectives

Among the multiple tumor-promoting characteristics of MDSCs, the capacity to suppress T-cell response remains a key hallmark. Given the complexity of the tumor immune microenvironment, it is not surprising that MDSCs are more than a T-cell suppressor and that their function is regulated on multiple levels. With the advances in phenotyping and functional assessment in recent years, a clearer view of the immunosuppressive nature of MDSCs has been achieved. Firstly, several novel suppressive mechanisms have been identified, which makes MDSCs a versatile suppressor. Secondly, the antigenicity of MDSC-mediated T-cell inhibition depends largely on the properties of the effector molecules utilized, since a different level and duration of intercellular contact is required; furthermore, differential suppressive potency and preferential mechanisms between subsets of MDSCs compartmentalize T-cell suppression in tumor-bearing host: immunosuppression is relatively weak and is antigen-specific in the periphery, while it is strong and is both antigen-specific and non-specific in the tumor milieu. Thirdly, the recognized targets of MDSCs have been extended from T cells to other immune cells, such as NK cells, DCs, and B cells, which broadens their suppressive spectrum and makes them suppressive in both innate and adaptive immunity. Lastly, in addition to clarification of their expansion and activation in the presence of tumor, the development and function of MDSCs are fine-tuned by several microenvironmental factors. With these characteristics unraveled, a pivotal role of MDSCs in the intricate network of immune suppression within the tumor microenvironment has been unveiled.

As a competent accomplice in carcinogenesis and disease progression, the correlation between MDSCs and tumor burden and disease stage is well-documented. For instance, a recent meta-analysis has shown for the first time that a high level of MDSCs is associated with shorter survival outcomes in patients with solid tumors and hematologic malignancies (228). This notion has two therapeutic implications. On the one hand, MDSCs have been regarded as an attractive target in cancer therapy. Various pre-clinical and clinical studies have shown promising benefits by targeting MDSCs, which can be achieved by depleting their quantity, blocking their trafficking, or inhibiting their immunosuppressive activity (5). On the other hand, due to their potent immunosuppressive capacity, MDSCs act as a major obstacle to natural anti-tumor immunity, hinder the efficacy of immunotherapy, and constitute an important mechanism for resistance. Accordingly, a high level of MDSCs predicts poor response to immune checkpoint inhibitor ipilimumab in metastatic melanoma patients (66). More importantly, it is rational to target MDSCs in combination with immunotherapy, which may yield a synergistic effect and delay, or even reverse, the occurrence of resistance (66). For instance, as compared to monotherapy, the efficacy of an immune checkpoint inhibitor or cancer vaccine is enhanced by combining with MDSC-targeted therapy in pre-clinical studies and clinical trials (66), and T cell-based immunotherapy efficacy is enhanced by inhibiting the trafficking of MDSCs (229). These benefits are associated with improved T cell-mediated immune response against tumor or increased antigen presentation capacity of DCs, probably due to the relieved inhibition imposed by MDSCs.

However, approaches to combat with MDSCs are still in their infancy, and there are several conundrums to be addressed. Considering their versatility and the complexity of microenvironmental regulation, the suppressive machineries of MDSCs are not likely to act simultaneously, but most probably function in a context-dependent manner. As a consequence, when we target the suppressive function MDSCs, it would be difficult to identify the most relevant target(s). Meanwhile, taking into account the indispensability of myelopoiesis in physiological processes and the phenotypic similarity between MDSCs and normal myeloid cells, it would be challenging to target MDSCs accurately without affecting the normal myeloid compartment.

Since they were firstly reported in the late 1970s and consensus on their nomenclature was reached in 2007, MDSCs as a group of suppressive myeloid cells have received increasing attention, and research on MDSCs is booming. Their roles in malignant and non-malignant settings are becoming clarified. With the effort in the past decade, an algorithm that includes phenotypic and functional, and, if possible, molecular criteria necessary to identify MDSCs was proposed in 2016 (4). This step-by-step approach aims to minimize ambiguity and helps to dissect the function of MDSCs in future studies. For instance, it may help us to better distinguish MDSCs from normal myeloid cells in the same host. Determining how to target MDSCs more precisely and efficiently relies, hopefully, on a better understanding of the development and suppressive nature of MDSCs. Lastly, more clinical trials are needed to validate the synergistic effect of MDSC-targeted therapy and cancer immunotherapy.

## Author Contributions

YY conceptualized the study, wrote the manuscript, and approved the final draft for publication. YY and CL prepared the figures. TL, XD, and AB reviewed and edited the manuscript. All authors contributed to the article and approved the submitted version.

## Conflict of Interest

The authors declare that the research was conducted in the absence of any commercial or financial relationships that could be construed as a potential conflict of interest.
